# Simulator Availability in Meeting the Requirements of United States Army Ophthalmology Individual Critical Task Lists

**DOI:** 10.7759/cureus.5209

**Published:** 2019-07-23

**Authors:** Christopher M Anthony, John H Boden, Gary L Legault

**Affiliations:** 1 Ophthalmology, Western University of Health Sciences, Lebanon, USA; 2 Ophthalmology, Madigan Army Medical Center, Tacoma, USA; 3 Ophthalmology, Brooke Army Medical Center, San Antonio, USA

**Keywords:** ictl, simulator, army

## Abstract

Introduction

Individual Critical Task Lists (ICTLs) are a list of requirements set forth by the United States Army which each soldier must fulfill to maintain competency in a specialty. By providing senior leadership objective criteria with which to evaluate the competency of each service member, ICTLs support commanders in ensuring that soldiers are mission ready and deployable. Board-certified ophthalmologists can meet ICTL requirements by demonstrating skills on an actual patient, a simulator, and/or cadaveric or live tissue. We sought to determine the availability of simulators that can be used to meet Army ophthalmology ICTL requirements.

Methods

We reviewed the current Army ICTLs for ophthalmologists. We performed an online search, as well as an extensive review of Pubmed, AccessMedicine, Academic Search Elite, Thieme, and ScienceDirect, to identify available simulators for each ICTL. We did not use any date or language restrictions in the electronic search for trials. We last searched the electronic databases on April 27, 2019.

Results

Army Ophthalmologists are required to maintain current status in 19 areas based on ICTLs established by the Critical Task Site and Selection Board. Eight of these requirements are not amenable to a simulation of any kind. Of the 11 remaining ICTLs, approximately 82% can be satisfied with a simulator alone based on current simulator availability. The remaining 18% of applicable ICTLs can be satisfied using cadaveric or live tissue training.

Conclusions

Army ophthalmologists can keep current with their ICTLs, and thus maintain mission readiness, by using either simulators or cadaveric or live tissues. This is particularly important for ophthalmologists who are either located in remote or austere locations without resources or areas with low surgical volumes. Several tasks are applicable to other medical specialties which can benefit from the same simulators.

## Introduction

Army Individual Critical Task Lists (ICTLs) are defined as “lists of tasks deemed critical by the Critical Task Site and Selection Board which must be performed to accomplish [one’s] mission duties and to survive in the full range of Army operations” and are described for every career in the Army [1]. By providing senior leadership objective criteria with which to evaluate the competency of each service member within his or her specialty, ICTLs help to maintain both the functionality and efficiency of the Army. Concerning board-certified ophthalmologists, the competency requirements established through ICTLs may be met by demonstrating particular skills on an actual patient, a simulator, and/or cadaveric or live tissue within a set time interval. According to the 2019 ICTL Master Workbook established by the Critical Task Site and Selection Board, Army Ophthalmologists are required to maintain current status or proficiency in 19 areas. Given that Army ophthalmologists may be stationed in locations without adequate opportunities for maintaining proficiency in specialty-related skills, and also taking into account the growing body of evidence suggesting that practice on simulators enhances operator skill and improves patient safety, we sought to determine the availability of simulators that can be used to meet Army ophthalmology ICTL requirements [2]. 

## Materials and methods

We reviewed the current Army ICTLs for ophthalmologists. We performed an online search, as well as an extensive review of PubMed, AccessMedicine, Academic Search Elite, Thieme, and ScienceDirect using words or phrases such as "ophthalmology", "simulators", "live tissue ophthalmology simulation", and "high fidelity ophthalmology simulation," to identify available simulators for each ICTL. We did not use any date or language restrictions in the electronic search for information. We last searched the electronic databases on April 27, 2019.

## Results

According to the 2019 ICTL Master Workbook established by the Critical Task Site and Selection Board, Army Ophthalmologists are required to maintain current status or proficiency in 19 areas which are listed in Table 1. Eight of these 19 areas are not amenable to simulation of any kind, including (1) maintaining board certification in ophthalmology, (2) maintaining current, unrestricted privileges in ophthalmology, clinical and surgical care including ophthalmic trauma, (3.1) completing an ocular trauma course once every four years or instructing two ophthalmic courses in prior four years, (3.2) completing or instructing orbital dissection course or Uniformed Services University of the Health Sciences (USUHS) Expeditionary Craniofacial Trauma Course within prior four years, (3.3) maintaining current Advanced Trauma Life Support (ATLS) qualification as either provider or instructor, (3.4) maintaining current Advanced Cardiac Life Support (ACLS) or equivalent qualification as either provider or instructor, (3.5) demonstrating familiarity of Ophthalmic Joint Trauma System (JTS) Clinical Practice Guidelines (CPG) through passage of a knowledge exam every three years, and (4.1) performing at least 75 surgical cases in the prior 12 months as primary surgeon or teaching attending. Of the 11 remaining areas, including (4.2) demonstrating experience in the management of open globes through management of at least one open globe injury (or equivalent surgical substitutions) (4.3) providing orbital and/or adnexal injury care, (4.4) managing patients with enucleation/evisceration eye injuries, (4.5) providing periocular and/or ocular burn care, (4.6) providing corneal, scleral, and/or anterior segment surgical trauma management, (4.7) performing lateral canthotomy, (4.8) performing orotracheal intubation, (4.9) performing needle decompression for pneumothorax (4.10) chest tube placement, (4.11) tourniquet placement, and (4.12) participating in at least one military training facility (MTF)-wide or post-wide mass casualty (MASCAL) event or exercise within the past 12 months, approximately 82% can be satisfied with simulators alone based on current commercial simulator availability. The remaining 18% of the ICTLs can be satisfied by using cadaveric or animal tissues. Table 2 lists the simulators available with the corresponding ICTLs.

**Table 1:** List of Army ICTLs

1.0	Maintain Board Certification in Ophthalmology
2.0	Maintain current, unrestricted privileges in Ophthalmology surgical and clinical care
3.0	Complete a training, qualification, knowledge exam
3.1	Complete the Ocular Trauma Course once every 4 years
3.2	Complete the Orbital Dissection Course once every 4 years
3.3	Maintain ATLS
3.4	Maintain ACLS
3.5	Complete knowledge exam every 3 years on Ophthalmic JTS CPGs
4.1	Perform 75 surgical cases within 12 months
4.2	Demonstrate experience in management of open globes
4.3	Provide orbital and/or adnexal injury care
4.4	Manage patients with enucleation/evisceration eye injuries
4.5	Provide periocular and/or ocular burn care
4.6	Provide corneal, scleral, and/or anterior segment surgical trauma management
4.7	Perform lateral canthotomy/cantholysis
4.8	Perform orotracheal intubation
4.9	Perform needle decompression for pneumothorax
4.10	Perform chest tube placement
4.11	Tourniquet placement
4.12	Participate in at least 1 MTF-wide or post-wide MASCAL event or exercise within the past 12 months

**Table 2:** List of Simulators for ICTLs

ICTL	Simulator commercially available exists (Y/N)	Cadaveric and/or animal tissue used (Y/N)	Example simulators	Approximate Cost in U.S Dollars
4.2: Open globe management	N	Y		
4.3: Orbital or adnexal injury care	Y	Y	Eye Lids Flex Orbit Accessory (Bioniko, Miami, FL), Ocular and Craniofacial Trauma Treatment Training System [3]	$100-$500
4.4: Enucleation/evisceration eye injury management	Y	Y	Exos Enucleation Simulator (Bioniko, Miami, FL)	$250
4.5: Periocular and/or ocular burn care	N	Y		
4.6: Cornea, sclera, anterior segment trauma management	Y	Y	Okulo BL5 Trainer, Cordelia Recovery Simulator (Bioniko, Miami, FL)	$75 - $500
4.7: Lateral canthotomy	Y	Y	Low Fidelity Model, SynDaver Lateral Canthotomy Trainer [4-5]	$8 - $750
4.8: Orotracheal intubation	Y	Y	Economy Adult Airway Management Trainer (Simulaids, Saugerties, NY)	$980
4.9: Need decompression	Y	Y	Tension Pneumothorax Simulator (Simulaids, Saugerties, NY), Pneumothorax Training Manikin (3B Scientific, Tucker, GA)	$600 -$650
4.10: Chest tube placement	Y	Y	Life/form Chest Tube Manikin (3B Scientific, Tucker, GA)	$1,700
4.11: Tourniquet placement	Y	Y	Life/form First Aid Arm (Nasco, Saugerties, NY), Tactical Operations Manikin (Innovative Tactical Training Solutions, Crestwood, KY)	$685 - $44,000
4.12: MASCAL event or exercise	Y	N	SimMan 3G (Laerdal Medical, Wappingers Falls, NY), METI Man HPS (CAE Healthcare, Sarasota, FL)	$96,000 - $200,000

## Discussion

Insult to the eye is incredibly common in traumatic incidents, especially in the military. During the first eight months of the Iraqi Insurgency in 2004, approximately 10% of the surgical patients admitted to the 31st Combat Support Hospital (CSH) suffered severe ocular or ocular adnexal injuries [6]. Considering the frequency and potentially devastating consequences of ocular injury in the deployed setting, ICTLs have been established for Army ophthalmologists to ensure that they maintain the skills necessary to manage ocular trauma. The presumed method to stay clinically competent and operationally ready was with an actual patient and surgical exposure. In recent years, however, ophthalmologic simulators have allowed specialists to safely and ethically acquire and maintain skills necessary for mission readiness, even when stationed at locations where access to adequate surgical volume may be limited [3]. We sought to determine how many ophthalmology ICTLs, and therefore what portion of mission-critical skills, could be maintained by non-biological tissue simulators alone without the need for actual patient exposure. 

**Figure 1:**
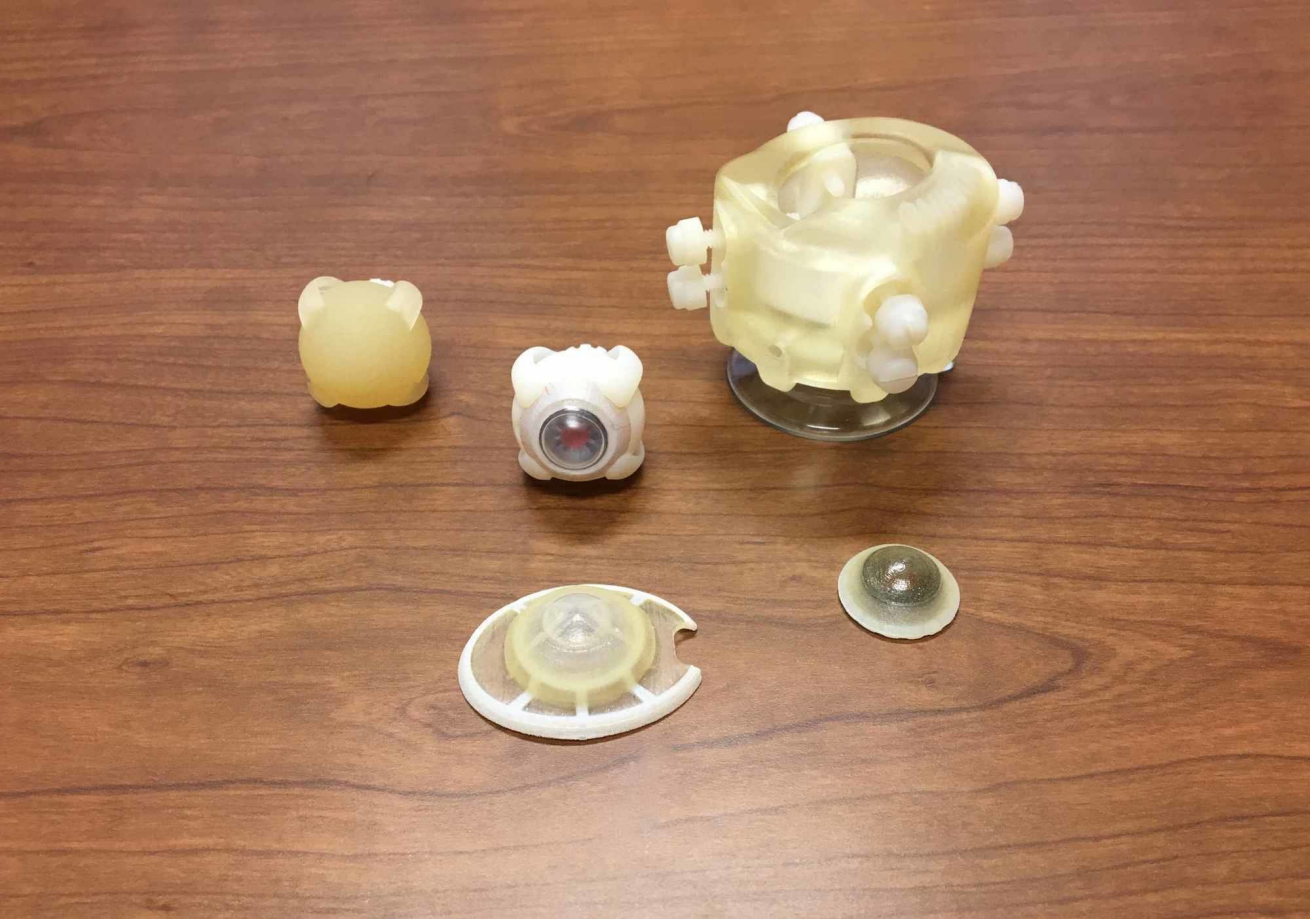
Various Ophthalmological Simulators

**Figure 2:**
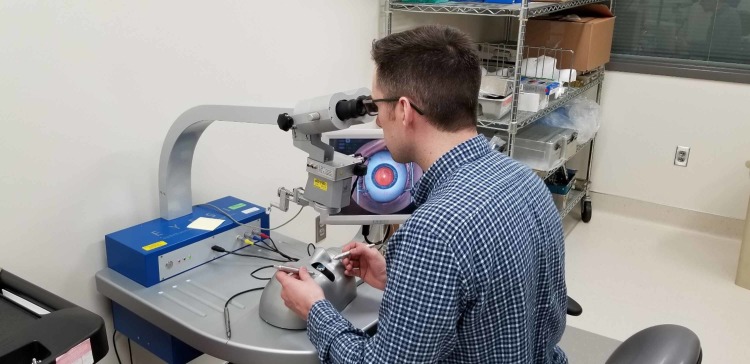
Medical Student Working on the Eyesi® Simulator (VRmagic, Mannheim, Germany)

We found that approximately 82% of the applicable ICTLs can be satisfied with simulators based on current commercial simulator availability. The remaining 18% of the ICTLs can be satisfied by using cadaveric or animal tissues. Therefore, Army ophthalmologists can keep current with their ICTLs and maintain full mission readiness through simulation alone. Figures 1, 2 illustrate common ophthalmology simulators. The paradigm shift within military medicine towards training with non-biologics corresponds well with a recent directive from the Department of Defense calling for a “reduction in the use of animals for medical education training when alternative methods produce scientifically or educationally valid or equivalent results [3].” 

With the recent mandate from the Department of Defense to reduce the use of animals for medical training in favor of alternative methods, it is important to understand the potential advantages of non-biological simulators. These may include the promotion of patient safety, cost savings, the ability to customize teaching opportunities to individual learner needs, acquiring a range of surgical skills without compromising patient safety or comfort, increased exposure to complex and life-threatening events that may be lacking due to short hospital stays or restriction in work hours, and “warming up” before starting an operation [4-5, 7]. Each of the aforementioned items is particularly applicable to deployed medical personnel who may lack the time, equipment, or patient volume necessary to maintain an appropriate level of surgical skill.

Although our study shows that simulators are available to allow Army ophthalmologists to meet the skill requirements defined within ICTLs, it does not compare the effectiveness of simulated models vs. live tissue models or actual patients in helping learners to acquire and retain skills. According to Quick, “Despite technological advancements leading to the development of complex and interactive high-fidelity and virtual reality simulators, they lack the realism that can be portrayed with live tissue models. Learner engagement is a key concept when debating the effectiveness of simulation in education, and live tissue has been shown to increase engagement substantially. Start-up costs are high, and facilities are few, however, the benefits of live tissue training cannot be overlooked” [8]. Additionally, our research does not address whether or not Army medical facilities, either domestically or abroad, actually own or have access to commercially available simulators. Further investigation into these areas may be beneficial not only for informing future training curriculum but also for improving patient safety and outcomes. 

## Conclusions

Army ophthalmologists can keep current with their ICTLs, and thus maintain mission readiness, by using a combination of simulators and cadaveric or live tissues. This is particularly important for ophthalmologists who are located in remote or austere locations where resources may be scarce or in areas with low surgical volumes. Several tasks are applicable to other medical specialties which can benefit from the same simulators.

## References

[1] (2019). Task analyst. https://usachcstraining.army.mil/analyst.

[2] Dawe S, Pena G, Windsor J, Broeders J, Cregan P, Hewett P, Maddern G (2014). Systematic review of skills transfer after surgical simulation‐based training. Br J Surg.

[3] Sykes S, Chou E, Mazzoli R, Pasternak J, Ryan D, Sia R, Colyer M (2018). Comparison of simulation-based versus live tissue-based ocular trauma training on novice ophthalmologists: repair of marginal eyelid laceration model. J Acad Ophthalmol.

[4] Herman L, Koch B, Benauer-Benning J (2017). A low fidelity model for teaching lateral canthotomy procedure. West J Emerg Med.

[5] Kong R, Kaya DP, Cioe-Pena E, Greenstein J (2018). A low fidelity eye model for lateral canthotomy training. Afr J Emerg Med.

[6] Mader T, Carroll R, Slade C, George R, Ritchey J, Neville S (2006). Ocular war injuries of the Iraqi insurgency, January-September 2004. Ophthalmology.

[7] Sachdeva A, Pellegrini CA, Johnson KA (2008). Support for simulation-based surgical education through the American College of Surgeons. World J Surg.

[8] Quick JA (2018). Simulation Training in Trauma. Mo Med.

